# Author's Responses to Peer Reviews of “Toward Human Digital Twins for Cybersecurity Simulations on the Metaverse: Ontological and Network Science Approach”

**DOI:** 10.2196/38587

**Published:** 2022-04-20

**Authors:** Tam N Nguyen

**Affiliations:** 1 Department of Management Information Systems University of Arizona Tucson, AZ United States

**Keywords:** human behavior modeling, cognitive twins, human digital twins, cybersecurity, cognitive systems, digital twins, Metaverse, artificial intelligence

*This is the author’s response to peer-review reports for “Toward Human Digital Twins for Cybersecurity Simulations on the Metaverse: Ontological and Network Science Approach.”*

## Round 1 Review

I am very grateful to the reviewers for their constructive comments regarding my paper [1]. I diligently considered the comments and made these key changes:

### Reviewer Z [2]

1. The author states that this paper proposes an application of Digital Twins (DT) and Human Digital Twins (HDT) for the first time. This is not exact, as, in the last 2 years, there have been some approaches to the use of DT in cybersecurity.

The author should include some of these ideas in the literature review.[...]

Response: I removed the claim that Reviewer Z had mentioned for added clarity. I did not discuss the recommended literature that does not involve Human Digital Twins (HDT) or any cognitive feature. Instead, I discuss 5 papers about autonomous agents (cognitive twins) for cybersecurity in the *Prior Work* subsection. I also emphasized the differences between those autonomous agents and HDTs in the *Conclusions* section to refocus the audience’s attention on HDTs for cybersecurity—the paper’s main research topic.

2. In the literature review, the author should add a definition of DT and HDT, how HDT surges from the concept of DT, a comparison between both techniques, and finally a list of the main uses of DT and HDT.

Response: I made clearer the definitions of DT and HDT and added a brief definition of Metaverse in the *Introduction* section and the *Backgrounds on HDTs* subsection. I also point the audience to the *Prior Work* subsection for a list of most relevant use cases for cognitive twins (autonomous agents).

3 and 4. In the literature review, the author claims that there is no grounded vision of the power of DT and HDT. In addition to the fact that, as I mentioned before, there are already applications of DT to cybersecurity, nothing is mentioned about proactive cyber defense existing techniques. What can DT and HDT add to the existing techniques?

The author states that the framework targets the cognitive process of a malicious actor as an HDT within a DT system. What is the purpose of this? The author must explain why these decisions were made.

Response: I added two paragraphs (the second and fourth paragraphs) in the *The General Landscape* subsection and discuss use cases of cognitive twins in the *Prior Work* section. Those details, the *Backgrounds on HDTs* and the Cybonto Conceptual Framework for a total of 15 related references should give the audience a good sense of the needs, benefits, and basic mechanisms of HDTs for cybersecurity vision.

5 and 6. Regarding Table 1, how was the total score calculated? There should be a description of every item. How was the score of every item calculated? An explanation is necessary.

Related to the above, it is good to have all the information in GitHub, but, at least a brief and clear description of the obtention of cybersecurity-related behavioral theories, and another description of the ontology should be provided in the manuscript or in a Multimedia Appendix.

Response: A paragraph was added for a brief explanation of table metrics calculations. I also added the Cybonto main hierarchies figure (Figure 1). Figure 1 and figure 2 should be able to convey the Cybonto core details. I emphasized the need of visiting the GitHub repository for the most up-to-date of all the artifacts at least two times in the paper. I believe it is the best way for the JMIR readership. For example, it is best to download the latest Cybonto and browse it interactively via Protégé.

7. An explanation of Figure 1 is needed.

Response: The original Figure 1 (Cybonto Conceptual Framework figure) was explained in the entire “Expanding the Vision With The Cybonto Conceptual Framework” subsection.

8. Without a clear description, the rest of the paper, although interesting, is difficult to follow.

Response: I added new sections and reformatted the entire paper per JMIR author guidelines. I hope that makes the paper easier to follow.

9. In broad terms, I understand the goal of the ontology, but it is so abstract that it is difficult for me how to apply it to proactive cyber defense. Some examples would be welcome. 

Response: I added the “Prior Work” subsection and discuss interesting use cases of applying human-like agents (cognitive twins and autonomous agents) in the physical-cyber security domain.

10. Last, a general comment: this is the Journal of Medical Internet Research. Though other topics are welcome, and it is clear that security is capital in the medical field, some particular comments about cybersecurity in the medical field would be desirable.

Response: I reworked the “Conclusions” section with an emphasis on the potential use of Cybonto in virtual patients for applied psychology training, automatic behavioral annotations, and analysis of Electronic Health Records, and virtual agents for community psychology experiments.

11. In the introduction, the author states that “incredibly,” HDT offers the capability of running large-scale simulations. Why “incredibly”?

Response: I removed “incredibly.”

12. In the introduction, the author claims that “Analyzing the Cybonto ontology informed the Cybonto conceptual framework.” I do not understand this sentence.

Response: I reworked the “Introduction” section and removed the confusing sentence.

13. The author defines the in-group environment acronym as IGE, but it appears as IEG in the rest of the paper.

Response: I corrected the typographical errors.

## Round 2 Review

### Reviewer Z

1. In Table 2, what are PR, EC, BC, and DC?

Response: PR stands for “Page Rank,” EC stands for “Eigenvector centrality,” BC stands for “betweenness centrality,” and DC stands for “degree centrality.” I added table footnotes to clarify this information.
